# First Report on the Acoustic Signals of Lahille’s Bottlenose Dolphins in Argentina

**DOI:** 10.3390/ani16050822

**Published:** 2026-03-06

**Authors:** Gisela Giardino, Agustina Macchi, Margherita Silvestri, Franck Malige, Ricardo Bastida, Mauricio Soto-Gamboa, Iván A. Hinojosa, Diego Rodríguez, Ignacio Rabinovich, Herve Glotin, Julie Patris

**Affiliations:** 1 Instituto de Investigaciones Marinas y Costeras (IIMyC), Facultad de Ciencias Exactas y Naturales, Universidad Nacional de Mar del Plata(UNMdP)-CONICET, Mar del Plata 7600, Argentina; agusmacchi.1995@gmail.com (A.M.); bastidaricardo@gmail.com (R.B.); dhrodri@gmail.com (D.R.); ignacio.rabinovich@yahoo.com.ar (I.R.); 2 CIAN—International Center of Artificial Intelligence in Natural Acoustics, Université de Toulon, CEDEX 9, 83041 Toulon, France; franck.malige@univ-tln.fr (F.M.); herve.glotin@lis-lab.fr (H.G.); julie.patris@univ-amu.fr (J.P.); 3 Instituto de Ciencias Ambientales y Evolutivas, Facultad de Ciencias, Universidad Austral de Chile (UACh), Valdivia 5090000, Chile; margherita.silvestri22@gmail.com (M.S.); mrsoto@uach.cl (M.S.-G.); 4 DYNI Team, LIS Laboratory, CNRS UMR 7020, Université de Toulon, CEDEX 9, 83041 Toulon, France; 5 Departamento de Ecología, Facultad de Ciencias, Universidad Católica de la Santísima Concepción (UCSC), Concepción 4090541, Chile; ihinojosa@ucsc.cl; 6 Centro de Investigación en Biodiversidad y Ambientes Sustentables (CIBAS), Universidad Católica de la Santísima Concepción (UCSC), Concepción 4090541, Chile; 7 Centro de Ecología y Manejo Sustentable de Islas Oceánicas (ESMOI), Departamento de Biología Marina, Universidad Católica del Norte, Coquimbo 1710164, Chile; 8 Centro de Investigación Oceanográfica COPAS COASTAL, Universidad de Concepción, Concepción 4070409, Chile; 9 Département de Physique, Aix-Marseille University, 13013 Marseille, France

**Keywords:** *Tursiops gephyreus*, acoustic repertoire, passive acoustic monitoring, Bahía Blanca Estuary, echolocation, whistles, bray calls, Argentina

## Abstract

This study provides the first characterization of the acoustic signals emitted by Lahille’s bottlenose dolphins (*Tursiops gephyreus*) in Argentine waters, establishing a baseline for the species’ southern distribution limit. Passive acoustic monitoring was conducted in the inner channels of the Bahía Blanca Estuary using a broadband hydrophone and F-POD detectors. We documented a diverse acoustic repertoire consisting of echolocation clicks, whistles, bray-calls, and chirps. Acoustic presence was predominantly diurnal and showed a marked synchronization with the tidal cycle, with peak activity occurring during the ebbing tide. Whistles had a mean center frequency of 8.1 kHz, showing spectral overlap with other regional populations in the Southwestern Atlantic. Echolocation clicks were broadband, with peak frequencies ranging from 18 to 127 kHz and a mode around 40 kHz. These results suggest that dolphin movement in the estuary’s internal channels is structured by hydrodynamics, likely reflecting foraging opportunities associated with tidal flow. Given the endangered status of this species, these data establish a technical framework for non-invasive monitoring and habitat-use assessments in complex estuarine environments.

## Simple Summary

Lahille’s bottlenose dolphin is the most endangered small cetacean in Argentina. While it was once common along the northern coast of Buenos Aires, the population collapsed in the 1980s and 1990s due to overfishing and pollutants, leaving only small, isolated groups today. These fragmented and small populations are difficult to study using traditional visual studies. In this study, we used passive acoustic monitoring—underwater sound recorders—to detect dolphins in the turbid inner channels of the Bahía Blanca Estuary. We provide the first detailed description of the species’ acoustic signals in Argentina, including echolocation clicks and communication whistles. Our results reveal that dolphin activity is predominantly diurnal and strongly synchronized with the tidal cycle, particularly during the ebbing tide. These findings suggest that dolphins coordinate their movements with water flow to optimize foraging in a complex environment. These findings establish a preliminary baseline for non-invasive monitoring and support the development of long-term conservation strategies for this vulnerable population in environments heavily influenced by human activities.

## 1. Introduction

The Lahille’s bottlenose dolphin (*Tursiops gephyreus*) occurs along approximately 3500 km of coastline in the Southwestern Atlantic, from Paraná, Brazil, (around 25°S; [1]) to Chubut, Argentina (around 46°S; [2,3]). It inhabits shallow coastal waters and is primarily distinguished by its larger body size, as well as by morphological and genetic differentiation from the offshore form (*Tursiops truncatus*), which occurs in the same area [1,4,5]. Although the former has occasionally been observed in offshore waters, its distribution generally remains within a maximum range of approximately 3 km from the coast [6,7].

Historically, *Tursiops gephyreus* populations along the northern coast of Buenos Aires and Uruguay maintained stable densities throughout the first half of the 20th century. However, a gradual decline began in the mid-1970s, culminating in a sharp collapse during the 1980s. By the 1990s, the species had almost entirely disappeared from the northern coast of Buenos Aires Province [8,9]. This population collapse has been attributed to intense coastal overfishing and high concentrations of pollutants, which likely reduced the reproductive potential of the species [7,10,11]. This regional disappearance coincided with an intense increase in industrial fishing effort, which led to the overexploitation of key prey species, such as the whitemouth croaker (*Micropogonias furnieri*) and the stripped weakfish (*Cynoscion guatucupa*) [11].

At present, a geographic gap of approximately 700 km separates the subpopulations of southern Brazil–Uruguay from those along the Argentine coast, limiting demographic and genetic exchange. In Argentina, the total population of Lahille’s bottlenose dolphin is estimated at fewer than 200 individuals [9]. Currently, the species occurs in three main coastal regions: Bahía Blanca and Bahía San Blas (southern part of Buenos Aires Province), Bahía San Antonio (Río Negro), and the coast of Chubut Province. Abundance estimates indicate approximately 34 individuals in Chubut [12] and around 83 individuals in Río Negro [13]. In southern Buenos Aires Province, including Bahía Blanca, no dedicated abundance estimates are available; however, photo-identification data collected between 2010 and 2016 suggest that the number of individuals is low and does not exceed 50 dolphins [9]. Given the small size and isolation of these subpopulations, low encounter rates, and the disappearance of the northern Buenos Aires group, *T. gephyreus* is currently considered the most endangered small cetacean along the Argentine coast and is categorized as Endangered by the IUCN Red List [14]. In this context, non-invasive monitoring approaches are particularly important. Passive acoustic monitoring offers a valuable tool for detecting presence, assessing habitat use, and supporting long-term population monitoring in small and elusive coastal dolphin populations [15,16].

In all bottlenose dolphins (*Tursiops* spp.), acoustic communication plays a central role in social organization, with frequency-modulated tonal whistles being fundamental for underwater communication, individual recognition, and group cohesion (e.g., [17,18,19,20]). As a result, whistle characteristics such as frequency, duration, and contour shape have been widely used to investigate geographic and population-level variation within the genus (e.g., [21,22,23]).

The vocal repertoire of Lahille’s bottlenose dolphins includes narrowband tonal whistles and broadband echolocation clicks emitted in trains [23]. Whistles are continuous, frequency-modulated signals typically below 40 kHz, exhibiting substantial variation in duration, frequency range, and contour complexity. Studies conducted in southern Brazil and Uruguay have reported whistles ranging from tens of milliseconds to over two seconds in duration, with most energy concentrated below 15 kHz and a predominance of simple contours, although more complex modulation patterns also occur [16,18]. Echolocation clicks consist of short-duration broadband pulses, usually arranged in trains, with peak frequencies commonly reported around 50–60 kHz and associated with navigation and prey detection [16].

In addition to whistles and echolocation clicks, complex pulsed signals composed of rhythmic multi-element sequences have been described in bottlenose dolphins, primarily in coastal populations of Lahille’s bottlenose dolphins and often in feeding or social contexts [24,25,26,27]. However, the occurrence, structure, and behavioural context of these signals have not been systematically assessed in *Tursiops gephyreus*, and their contribution to the acoustic repertoire of this species remains poorly documented.

Although existing studies indicate broadly similar acoustic characteristics among southern Brazilian and Uruguayan populations of Lahille’s bottlenose dolphins, fine-scale variation has been reported, likely influenced by social structure, behaviour, and local environmental conditions [23]. To date, no comparable acoustic studies have been conducted in Argentina, leaving the southernmost limit of the species’ distribution acoustically uncharacterized. Therefore, the aim of this study is to provide the first description of the acoustic signals produced by Lahille’s bottlenose dolphins in Argentine waters, based on passive acoustic recordings collected in the Bahía Blanca Estuary.

## 2. Materials and Methods

### 2.1. Study Area

**Figure 1:**
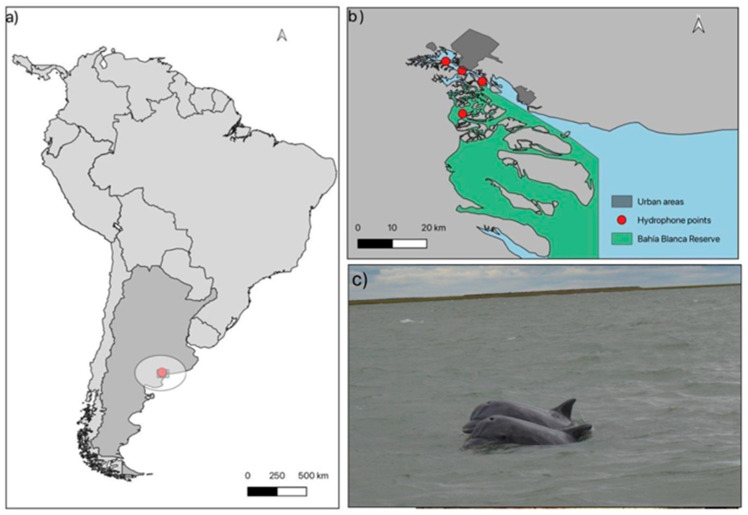
Study area showing Argentina (**a**) and the Bahía Blanca Estuary (**b**), with passive acoustic monitoring sites, including “Los Pichones” (red dot). Panel (**c**) shows Lahille’s bottlenose dolphins (*Tursiops gephyreus*) in the Bahía Blanca Estuary. Base map from the OpenStreetMap project.

The present study was conducted in the Bahía Blanca Estuary, located in the southwest of Buenos Aires Province, Argentina (Figure 1). This estuary is a mesotidal, semidiurnal system dominated by tidal forcing and characterized by strong vertical mixing under typical conditions. The water column is generally vertically homogeneous due to low freshwater input, with only occasional and short-lived stratification following episodic flood events [28].

### 2.2. Instruments and Deployments

During September 2025, acoustic recordings were collected as part of the French–Chilean–Argentine project (Detection and AI Classification of the Biosonar of Dolphins and Porpoises in the Southern Cone, IRP CNRS). As part of this project, four sets of passive acoustic instruments were deployed across internal tidal channels of the Bahía Blanca Estuary. One of these instrument sets was deployed in an inner channel sector locally known as “Los Pichones” (Figure 1). At this site, an autonomous broadband hydrophone (SoundTrap ST300HF, Ocean Instruments, Auckland, New Zealand) was deployed together with an FPOD (Chelonia Ltd., Mousehole, UK). The SoundTrap recorded continuously at a sampling rate of 576 kHz with 16-bit resolution and a sensitivity of −176.4 dB re 1 V/μPa, allowing the simultaneous detection of whistles and broadband echolocation clicks.

The hydrophone was housed in a protective cage and positioned at water depths between 6 and 8 m, depending on the tidal stage at the deployment site. Reported current velocities in the Principal Channel can reach values on the order of 0.8–0.9 m s^−1^ during mid-ebb conditions, while lower and more variable velocities occur during flood stages [29,30].

Only the acoustic recordings obtained at the Los Pichones site were used in the present study, as Lahille’s bottlenose dolphin signals were detected exclusively at this location.

The F-PODs automatically detect and classify echolocation clicks into four categories based on their spectral and temporal characteristics: “NBHF” (narrowband high-frequency clicks), “Other cetaceans” (broadband clicks from non-NBHF odontocetes), “Sonar” (likely anthropogenic sources), and “Unclassed” (ambiguous or overlapping signals) [31,32,33].

In the Bahía Blanca Estuary, narrowband high-frequency (NBHF) clicks are primarily produced by franciscana dolphins (*Pontoporia blainvillei*), which emit high-frequency clicks centered above 100 kHz [34,35], whereas broadband echolocation clicks classified as “Other cetaceans” correspond to Lahille’s bottlenose dolphins (*Tursiops gephyreus*). These are the only two small odontocete species regularly occurring in the area [36], allowing a reliable interpretation of FPOD classifications at the species level.

Recordings from the autonomous hydrophone (Sountrap ST300) were selected based on the time period in which the FPOD registered positive dolphin detections. Hydrophone sensitivity was factory calibrated by the manufacturer (Ocean Instruments) on 29 March 2021, following standard manufacturer procedures.

For each detection event, a minimum of five minutes before and five minutes after the detection were extracted for detailed inspection. Measurements were conducted in Raven Pro 1.6 (Hann window, 512).

### 2.3. Statistical Analysis

To evaluate diel and tidal patterns in acoustic detections of Lahille’s bottlenose dolphins, generalized additive models (GAMs) were fitted using the *mgcv* package in R version 4.4.0 [37]. The number of dolphin detection-positive minutes per hour (DPM) was used as the response variable.

Hour of day and tidal height were included as explanatory variables and modelled as smooth terms. Hour of day was treated as a circular variable and fitted using a cyclic cubic regression spline to account for the continuity between 23:00 and 00:00 h, whereas tidal height was included as a continuous smooth term.

Models were fitted assuming a Poisson error distribution with a log link function and parameters were estimated using restricted maximum likelihood (REML). The basis dimension (k) for each smooth term was set to 10 and evaluated using diagnostic checks. The model was specified as

(1) $$log(DPM)=\beta_{0}+s(Hour)+s(Tidal\;height)$$

where $\beta_{0}$ is the intercept and s () denotes smooth functions that model non-linear relationships between the response variable and the explanatory variables.

Model assumptions and adequacy were assessed through inspection of residuals, convergence diagnostics, and checks of basis dimension using *gam.check* function from the *mgcv* package. The degree of association between hour of day and tidal height was evaluated using Pearson’s correlation coefficient.

All statistical analyses were performed in the R programming environment [38].

### 2.4. Vocalization Classification and Measurements

(1) Echolocation clicks

Echolocation click parameters were analyzed for Lahille’s bottlenose dolphins recorded in the Bahía Blanca Estuary. An initial set of 1083 clicks, previously selected from a single high-quality recording (Audio S1) obtained with a SoundTrap autonomous hydrophone (sampling rate: 576 kHz), was analyzed using custom scripts implemented in GNU Octave. This recording was selected based on its high signal-to-noise ratio and the presence of clearly defined echolocation click trains. For each click, a 1 ms signal window centered on the waveform maximum was extracted.

A suite of acoustic parameters was computed, including signal-to-noise ratio (SNR), inter-click intervals (ICI), click duration (rms, −10 dB and −20 dB criteria), peak and centroid frequencies, and bandwidths measured at multiple thresholds (rms, −3 dB, −10 dB and −20 dB), following the definitions provided by Malige et al. [39].

For the purposes of this study, only clicks with SNR > 20 dB were retained, resulting in a final dataset of 864 high-quality clicks. From this subset, ICI, peak frequency, −10 dB duration, and rms bandwidth were selected for quantitative analyses and for comparison with previously published datasets.

(2) Whistles and chirps

Whistles were defined as tonal, frequency-modulated signals with harmonic structure, typically lasting from several hundred milliseconds to several seconds. Whistles were inspected visually and aurally in Raven Pro 1.6 [40]. Only signals with a clear tonal contour were included, whereas whistles overlapping with other vocalizations, clipped signals, or those with unclear start or end points were excluded, following the criteria established in Macchi et al. [41]. Individual whistles were treated as separate units if separated by at least 200 ms from other vocalizations [42]. Whistles were graded according to signal-to-noise ratio (SNR) as: (1) faint but visible, (2) clear and unambiguous, and (3) prominent and dominant; only whistles graded 2 or 3 were measured [43].

Chirps were considered a subtype of whistles and were defined as short-duration tonal signals with clear frequency modulation, typically appearing as comma-shaped traces in time–frequency representations. Chirps generally lasted between 0.01 and 0.25 s and often exhibited upward or downward frequency sweeps [20,44,45,46]. Unlike echolocation clicks, chirps exhibited continuous tonal structure rather than impulsive broadband energy.

Acoustic parameters of whistles and chirps were extracted following Lima et al. [23] and Macchi et al. [41], based on the Raven Pro 1.4 User’s Manual [47], to allow direct comparison among studies. Parameters and units were:

- Duration 90% (s): interval containing 90% of the energy of the signal.
- Minimum frequency (MINF; Hz): lowest frequency within the contour.
- Maximum frequency (MAXF; Hz): highest frequency along the contour at −10 dB relative to the peak.
- Delta frequency (DF; Hz): difference between MAXF and MINF.
- Center frequency (MF; Hz): frequency centroid, or mean spectral frequency weighted by amplitude.
- Peak frequency (Hz): frequency with the highest energy within the selection.
- Bandwidth (BW90; Hz): frequency range encompassing 90% of the signal energy.

For chirps, duration, minimum frequency, maximum frequency, and peak frequency were determined whenever possible [20,42].

(3) Bray calls sequences

Bray calls were defined as sequences of acoustic elements composed of identifiable components such as squeaks (SQ), grunts (GR), and pops (POP) [48]. Elements were considered part of the same sequence if separated by less than one minute [24]. Each element within bray-call sequences was analyzed individually.

Parameters measured for each element were following the criteria of Luís et al. [26]; Pace et al. [48] and Connor & Smolkerlker, [49]. Duration was measured for all elements. Frequency parameters were extracted depending on the element type and only when they could be reliably measured. For SQ elements, minimum, maximum, and peak frequencies were determined, with spectral features extracted from the dominant harmonic sideband [50]. For GR elements, maximum and peak frequencies were measured when clearly identifiable, while minimum frequency could be determined in only one case. For POP elements, only peak frequency was measured [48,49].

Additionally, total sequence duration and the time intervals between consecutive components were quantified for all sequences. Isolated elements identical to those found in bray calls but not forming a sequence were classified separately as single occurrences.

Signal detection and preliminary classification for bray calls and whistles (including chirps) were conducted in Audacity [51], whereas all quantitative acoustic measurements were performed in Raven Pro. To visualize and annotate bray-call sequences, spectrograms were generated in R using a 4372-point window and 87.5% overlap, with a frequency range limited to 0–30 kHz. The window length was increased relative to other analyses to improve frequency resolution, allowing clear identification of individual POP and SQ elements composing the bray calls.

## 3. Results

### 3.1. Acoustic Presence and Temporal Patterns

Between 23 and 29 September 2025, acoustic data from the Los Pichones inner-channel station (Bahía Blanca Estuary, Argentina) were analyzed. The FPOD recorded a total of 8657 min (144.3 h) of monitoring effort. Lahille’s bottlenose dolphins were acoustically detected during 0.45% of the total FPOD recording effort, corresponding to approximately 39 min of detections. Dolphin acoustic activity was characterized by short-duration encounters, with 90% of detection-positive minutes (DPMs) occurring in isolated 10 min intervals rather than continuous hours of presence.

**Figure 2:**
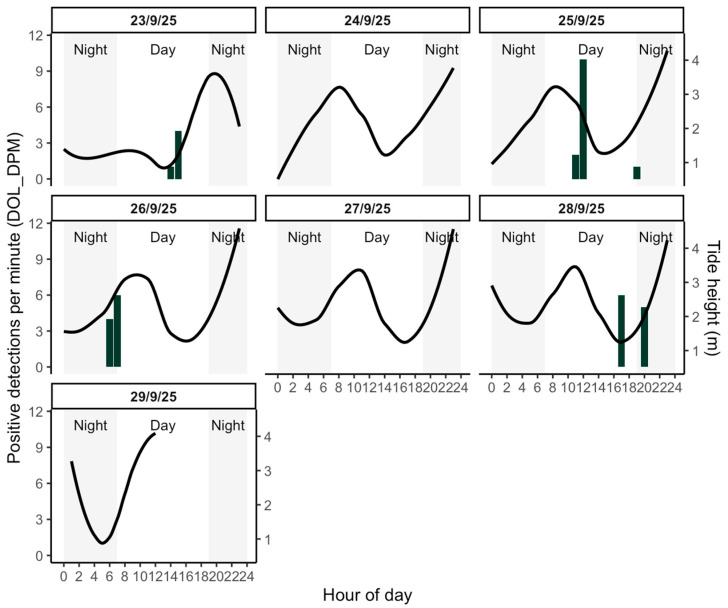
Diel variation in acoustic detections of Lahille’s bottlenose dolphin in the Bahía Blanca Estuary, Argentina, between 23 and 29 September 2025. Green bars represent positive detections per minute per hour (DOL_DPM). The black smoothed curve shows tidal height (m), displayed on a secondary y-axis. Light grey shaded areas indicate nighttime periods (before 07:00 and after 19:00 h). Each panel corresponds to a single sampling day.

Acoustic activity of *Tursiops gephyreus* in the inner channels of the estuary followed a non-random, intermittent pattern closely linked to tidal stages (Figure 2). Detections were predominantly recorded during daylight hours and were strongly associated with the ebbing tide and low water periods. The maximum acoustic presence was recorded on 25 September, with a peak of 10 DPMs at 12:00 h, occurring during a pronounced ebbing tide (dropping from 3.86 m to 2.23 m). Other significant peaks occurred during the early morning (e.g., 26 September, 07:00 h, 6 DPMs) and late afternoon (e.g., 28 September, 17:00 h, 6 DPMs), consistently matching periods of receding water. These visual trends were supported by Generalized Additive Models (GAM), which identified a significant non-linear relationship between dolphin presence and the hour of the day (edf = 6.58, *p* = 0.024). This exploratory model explained 72.1% of the deviance (adj. R^2^ = 0.57), reinforcing the observed diel pattern during the study period. Additionally, detection rates showed a significant non-linear association with tidal height (edf = 8.59, *p* < 0.001), despite a weak correlation between tide and hour of day (Pearson’s r = 0.20) (Table S1).

Narrowband high-frequency clicks consistent with those attributed to *Pontoporia blainvillei* were also detected during the monitoring period; however, these signals were not analyzed further in the present study.

### 3.2. Sound Productions

Lahille’s bottlenose dolphins in Bahía Blanca produced a diverse set of vocalizations, including whistles, echolocation clicks, burst-pulses, chirps, and bray calls.

Based on six days of continuous acoustic monitoring, the mean recording effort required to obtain a single detection differed markedly among vocalization types. On average, approximately 2.7 h of recording were required per whistle detection, 3.1 h per echolocation click detection, 62 h per bray call detection, and more than 70 h per chirp detection.

(1) Echolocation clicks

**Figure 3:**
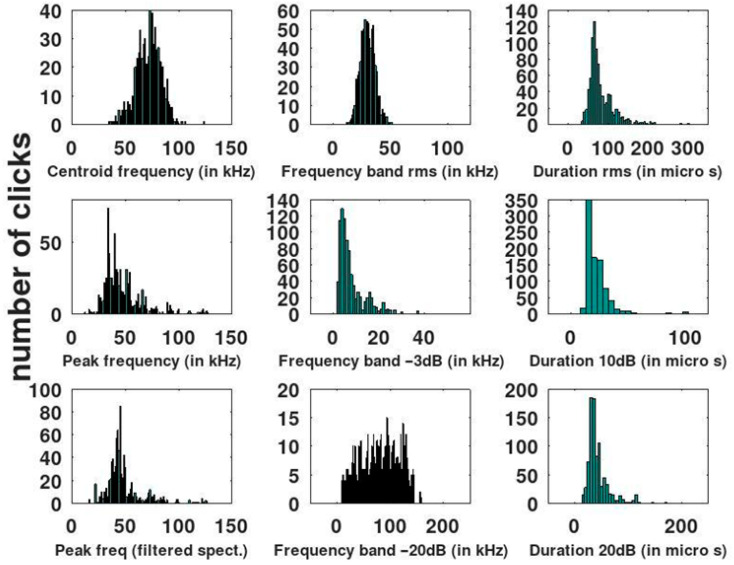
Histograms of echolocation click parameters of Lahille’s bottlenose dolphins recorded at Bahía Blanca Estuary. Only clicks with a signal-to-noise ratio (SNR) > 20 dB were included in the analysis (*n*= 864).

A total of 864 high-SNR clicks (SNR > 20 dB) from a single dataset file (S1) were analyzed (Figure 3). These clicks were grouped into high-amplitude click trains, characteristic of echolocation sequences produced at close range. Inter-click intervals (ICI) were (31 ± 19) ms, while peak frequency values proved highly variable, ranging from 18 kHz to 127 kHz. The peak frequency distribution showed a clear mode around 40 kHz, with a smaller proportion of clicks exhibiting values above 100 kHz. Click duration, measured using the Δt −10 dB criterion, was (22 ± 10) μs (range: 10 μs to 102 μs). Similarly, the RMS bandwidth of the clicks was (30 ± 6) kHz, with a range from 12 kHz to 51 kHz.

(2) Whistles and chirps

**Figure 4:**
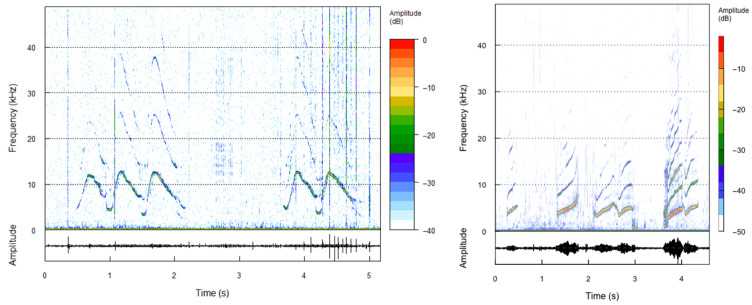
Representative whistle contours produced by a Lahille’s bottlenose dolphin in the Bahía Blanca Estuary, Argentina (two examples). The upper panel shows the spectrogram of frequency-modulated whistles, and the lower panel displays the corresponding oscillogram, illustrating its temporal structure.

A total of 163 whistles were analyzed (two examples are shown in Figure 4). In the Bahía Blanca Estuary, whistles showed a mean center frequency (MF) of 8.18 ± 1.94 kHz and a mean 90% bandwidth (BW90) of 6.85 ± 2.80 kHz. The mean minimum frequency (MINF) was 3.99 ± 1.39 kHz, while the mean maximum frequency (MAXF) reached 12.85 ± 3.77 kHz, resulting in a mean delta frequency (DF = MAXF–MINF) of 8.86 ± 3.93 kHz. The mean peak frequency was 8.17 ± 2.74 kHz. The mean whistle duration (DUR 90%) was 0.61 ± 0.44 s, with a range from 0.04 to 2.23 s (Table 1).

Six chirps were analyzed, although not all parameters could be identified for measurement. Chirp duration was (0.184 ± 0.111) (range: 0.076 s to 0.395 s), with minimum and maximum frequencies of (3.05 ± 1.31) kHz (range: 1.22 kHz to 4.57 kHz) and (8.02 ± 4.80) kHz (range: 3.11 kHz to 11.66 kHz), respectively. Peak frequency could only be determined for four of the six detected chirps, with a mean value of (5.70 ± 0.35) kHz (range: 5.34 kHz to 6.19 kHz) (Table 2).

(3) Bray calls sequences

Two bray call sequences were recorded, each consisting of a distinct sequence of acoustic elements. Across both sequences, three element types were identified: six Squeaks (SQs), three POPs, and seven Grunts (GRs) in total.

SQ elements had a duration of (0.167 ± 0.093) s (range: 0.035 s to 0.300 s), with minimum and maximum frequencies of (0.63 ± 0.35) kHz (range: 0.27 kHz to 1.24 kHz) and (1.38 ± 0.57) kHz (range: 1.00 kHz to 2.32 kHz), respectively, and a peak frequency of (3.89 ± 2.07) kHz (range: 0.49 kHz to 6.61 kHz). POP elements were longer, with a duration of (0.42 ± 0.11) s (range: 0.32 s to 0.53 s) and peak frequencies of (14.67 ± 2.31) kHz (range: 12 kHz to 16 kHz). GR elements had a duration of (0.248 ± 0.124) s (range: 0.16 s to 0.51 s). Maximum frequency values were (13.13 ± 4.86) kHz (range: 0.61 kHz to 20.72 kHz); only a single minimum frequency value could be determined (0.67 kHz). The peak frequency of GR was (5.91 ± 0.97) kHz (range: 4.78 kHz to 6.98 kHz). Peak frequency values for POPs were higher than those of the other components (14.67 ± 2.31) kHz, and in all cases the sequence consisted of 10 POPs.

**Figure 5:**
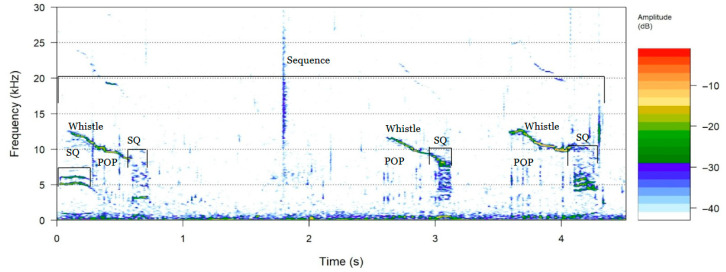
Example of a *Tursiops gephyreus* bray call sequence recorded in Bahía Blanca Estuary. The spectrogram was generated in R using an 8192-point window, 80% overlap, and a frequency range limited to 0–30 kHz. Distinct “POP” and “SQ” elements of the bray call sequence are indicated. Color levels represent amplitude in dB (from −42 to 2).

The first bray call sequence lasted 4.25 s and displayed an alternating SQ–POP–SQ–POP–SQ–POP–SQ pattern, with POPs reaching peak frequencies of 12 kHz and 16 kHz. Inter-element intervals were highly variable, with the longest gap of 1.84 s occurring between an SQ–POP transition, although some elements partially overlapped (Table 2; Figure 5). The second call sequence lasted 2.953 s and displayed an alternating SQ–GR–GR–GR–GR–GR–GR–SQ–GR pattern. This sequence was dominated by consecutive GR elements (0.158–0.299 s), while the SQ elements showed broader frequency ranges (0.512–2.324 kHz and 1.239–1.826 kHz). Intervals between GR elements were short and consistent (0.076–0.146 s) (Table 2; Figure 5).

In addition, a small number of isolated vocal elements not associated with bray-call sequences were detected. These included one POP element (duration: 0.004 s; peak frequency: 13.5 kHz), one GR element (duration: 0.040 s), and two SQ elements with durations of 0.176 s and 0.231 s. For these SQ elements, minimum frequencies ranged from 0.42 kHz to 0.99 kHz, maximum frequencies ranged from 0.75 kHz to 1.42 kHz, and peak frequencies were approximately 2.7 kHz.

## 4. Discussion

This study provides new information on the acoustic presence and temporal patterns of Lahille’s bottlenose dolphins (*Tursiops gephyreus*) in the Bahía Blanca Estuary (Argentina), contributing baseline data on their vocal activity in a dynamic coastal environment influenced by natural and anthropogenic factors.

### 4.1. Acoustic Presence and Temporal Patterns

Acoustic detections of *Tursiops gephyreus* in the Bahía Blanca Estuary were predominantly diurnal, with peak activity during mid-morning and late afternoon and almost no detections at night. While similar diurnal patterns occur in other bottlenose dolphin populations, there is significant regional variability within the genus. For instance, *Tursiops truncatus* in Florida exhibits crepuscular peaks [52], whereas Mediterranean populations show increased biosonar activity during nocturnal and mid-morning periods [53,54]. In contrast, the activity in Bahía Blanca appears strictly synchronized with local environmental forcing agents, reinforcing the idea of high behavioral plasticity in these estuarine dolphins.

Our results indicate that Lahille’s bottlenose dolphin’s presence in the internal channels is specifically associated with outgoing tides (ebb phase). This suggests a strategic use of the estuary’s marked current asymmetry, where ebb currents are significantly stronger than flood currents [55]. Dolphins may be optimizing energy expenditure by coordinating their movements with receding waters or targeting prey that becomes concentrated within the channels as the tide retreats from the expansive intertidal flats.

The association between dolphin detections and tidal height is consistent with previous studies conducted in Argentine waters, in Bahía San Antonio [56]. In contrast in this area bottlenose dolphins preferentially used shallow and intertidal habitats during high tide, likely in response to increased foraging opportunities as prey becomes accessible in newly inundated areas. In the Bahía Blanca Estuary during high tide, the water level covers extensive tidal flats and marshes—which characterize the inner-channel stations—with a mean depth of 0.5 to 1 m [30]. This inundation likely triggers a landward movement of prey, which dolphins follow into the shallower areas of the Principal Channel. Similar tidal-related movements have been reported for bottlenose dolphins in other coastal systems, where tidal flow influences short-term habitat use and prey availability (e.g., [57,58,59]).

Such variability supports the view that diel patterns of echolocation activity in bottlenose dolphins are context-dependent rather than species-specific, reflecting local ecological conditions such as prey availability and habitat structure. As an exploratory approach, our use of generalized additive models suggests that this acoustic presence is not random but structured by these variables. Nevertheless, a larger dataset—incorporating seasonal and inter-annual variations, would be necessary to increase statistical power and confirm whether these patterns represent a consistent, long-term behavioral strategy at a population level.

In addition, our data provide insight into the recording effort required to document different vocalization types in this area. The low detection rates of some signal types highlight the importance of sustained acoustic monitoring to obtain representative samples of the acoustic repertoire and activity patterns of *Tursiops gephyreus*.

### 4.2. Sound Productions

The acoustic repertoire of Lahille’s bottlenose dolphins in Bahía Blanca Estuary was diverse, including whistles, echolocation clicks, bray calls, and chirps.

The wide range of peak frequencies observed in this dataset, together with the pronounced mode around 40 kHz, reflects the diversity of click spectral properties recorded from Lahille’s bottlenose dolphins. Such variability is consistent with the known dependence of click peak frequency on the orientation of the emitting animal relative to the recording device, with off-axis clicks typically exhibiting lower and more variable peak frequencies than on-axis clicks [17,60,61,62]. The presence of a limited number of clicks with peak frequencies exceeding 100 kHz further supports this interpretation.

Click duration metrics obtained in this study are comparable to those previously reported for *Tursiops truncatus* in the Atlantic Ocean [63]. Although these authors used a different duration metric (D-duration), this measure has been shown to be closely related to the −10 dB duration criterion applied here [64], allowing for meaningful comparison across studies. The spectral consistency with other populations suggests that the fundamental echolocation parameters are well-conserved in *Tursiops gephyreus*. However, the high sediment load and the shallow, complex bathymetry of this estuary could influence signal propagation and reverberation levels, factors that should be further explored to understand fine-scale variations in click characteristics compared to open-water populations.

Similarly, rms bandwidth values measured in the present dataset fall within the range reported for other *Tursiops* populations [63], suggesting that the spectral characteristics of echolocation clicks recorded in Bahía Blanca Estuary are broadly consistent with those described elsewhere for the genus. Quantitative comparisons with click parameters reported for populations in Uruguay and Brazil were avoided due to methodological differences in data acquisition and parameter estimation.

Overall, whistle parameters fall within the ranges reported for Lahille’s bottlenose dolphins’ populations from southern Brazil [18,23] and Uruguay [16]. In particular, minimum and maximum frequencies largely overlap across regions, indicating broadly comparable whistle frequency ranges within the southwestern Atlantic coastal population. Moreover, the whistle parameters recorded in the inner channels of the Bahía Blanca estuary are consistent with our ongoing comparative studies of *Tursiops gephyreus* in the San Matías Gulf and under human care at Mundo Marino [65,66]. While some variation was observed in duration and bandwidth between habitats, the predominance of modulated and convex whistle shapes remained constant across all groups. Furthermore, the low whistle emission rate observed at our site is consistent with values reported for Lahille’s bottlenose dolphins in other coastal areas and also for animals under human care [65,66]. This suggests that a reduced investment in social signaling may be a stable behavioral trait of the species, regardless of environmental conditions such as water transparency or habitat type. Rather than a purely environmental constraint, this pattern could indicate that Lahille’s bottlenose dolphins relies more heavily on pulsed sounds and click trains for both navigation and social communication, a strategy similar to that of non-whistling cetacean species (e.g., [34,67,68]).

When the Argentine population is included, the observed overlap in frequency parameters suggests continuity rather than clear acoustic separation at this geographic scale. Small differences among studies should be interpreted with caution, as methodological factors—such as recording equipment, sampling rates, filtering settings, and parameter definitions—can strongly influence measured whistle characteristics. Previous studies have shown that whistle structure may vary with behavioral context, including cooperative foraging with artisanal fisheries [69], as well as with local anthropogenic conditions, such as boat traffic and group composition [70]. However, the present study was not designed to evaluate behavioral or contextual drivers of whistle variation. Standardized acoustic protocols and context-controlled recordings will be required to disentangle biological variability from methodological effects across regions.

Chirps were infrequent, short, and frequency-modulated, consistent with previous descriptions of chirps in bottlenose dolphins [20]. Similar patterns have been documented in Namibia [42] and the Adriatic Sea [71] for *Tursiops truncatus*, where chirps are rare and often occur alongside other vocalizations such as whistles and low-frequency sounds. These observations suggest that chirps are generally produced in specific social contexts or for short-range communication.

Two multi-element bray-call sequences were recorded, exhibiting alternating SQ, POP, and GR units. While the temporal patterns of individual elements were consistent with observations from Mediterranean *Tursiops* populations [26,48], the combination and ordering of elements were more variable, supporting previous reports of high structural plasticity. Such flexibility may serve multiple social functions, including foraging coordination or intra-group communication [25,48]. The relatively low occurrence and limited diversity observed here likely reflect the continuous passive monitoring approach, which captures vocalizations opportunistically rather than from actively tracked individuals.

## 5. Conclusions

The present provides the first acoustic characterization of *Tursiops gephyreus* in Argentine waters, documenting a diverse repertoire that includes whistles, echolocation clicks, and complex pulsed sounds. Our findings demonstrate that passive acoustic monitoring (PAM) is a highly effective, non-invasive tool for studying this endangered population in the turbid and high-energy environment of the Bahía Blanca Estuary.

The results reveal that dolphin presence in the inner channels is not random but structured by a combination of light cycles and tidal hydrodynamics. The marked synchronization with the ebbing tide suggests a strategic behavioral adaptation, likely to optimize energy expenditure or exploit foraging opportunities as prey becomes concentrated by receding waters. While these preliminary patterns require validation through longer-term, multi-seasonal datasets to account for potential site-specific or seasonal variations, they establish a critical baseline for the species.

Ultimately, this research provides the technical framework necessary to integrate acoustic data into regional conservation strategies. Understanding how *Tursiops gephyreus* utilizes these anthropogenically influenced channels is essential for developing effective management plans aimed at protecting the most endangered small cetacean in Argentina.

## Figures and Tables

**Table 1:** Mean ± standard deviation and minimum–maximum values of whistle acoustic parameters for *Tursiops gephyreus* from Uruguay, the Patos Lagoon Estuary, and the Tramandaí Channel in Brazil (Lima et al. 2020 [23]), and the Bahía Blanca Estuary, Argentina (this study). Frequency parameters include minimum frequency (MINF; kHz), maximum frequency (MAXF; kHz), delta frequency (DF = MAXF − MINF; kHz), and mean frequency (MF; kHz, frequency centroid). Duration (DUR; s) corresponds to the interval containing 90% of the energy of the whistle. Bandwidth (BW90; kHz) encompasses 90% of the energy of the whistle, and Peak Frequency (Peak Freq.; kHz) corresponds to the frequency with the highest energy within the whistle selection, which may differ from MAXF if the maximum amplitude occurs at a lower frequency.

Parameter	Bahía Blanca Estuary, Argentina (n = 163)	Uruguay (n = 42)	Patos Lagoon Estuary, Brazil (n = 100)	Tramandaí, Brazil (n = 100)
MINF (kHz)	4.0 ± 1.4 (0.9–8.9)	4.9 ± 1.6 (2.6–9.6)	4.5 ± 1.4 (1.8–7.6)	5.3 ± 1.6 (2.2–12.5)
MAXF (kHz)	12.9 ± 3.8 (5.8–38.4)	10.6 ± 3.5 (4.6–17.2)	12.4 ± 3.5 (4.0–21.1)	11.4 ± 2.3 (7.0–21.2)
DF (kHz)	8.9 ± 3.9 (2.4–34.4)	5.7 ± 3.8 (0.5–13.9)	7.9 ± 3.6 (0.6–17.6)	6.1 ± 2.6 (0.7–15.5)
MF/Center Freq. (kHz)	8.2 ± 1.9 (3.4–12.8)	7.0 ± 1.9 (3.5–10.6)	8.0 ± 2.1 (3.0–13.5)	7.6 ± 1.5 (5.3–13.6)
BW90 (kHz)	6.8 ± 2.8 (1.9–15.8)	—	—	—
Peak Freq. (kHz)	8.2 ± 2.7 (1.0–15.8)	—	—	—
DUR 90% (s)	0.6 ± 0.4 (0.04–2.2)	0.7 ± 0.5 (0.8, 0.06–2.0)	0.7 ± 0.4 (0.5, 0.08–1.8)	0.6 ± 0.5 (0.05–2.9)

**Table 2:** Temporal and spectral parameters (mean ± SD) of the acoustic elements identified in Lahille’s bottlenose dolphin vocalizations recorded in the Bahía Blanca Estuary, Argentina. Parameters are shown for Squeaks (SQs), Grunts (GRs), and POP elements composing bray calls, as well as for isolated chirps. n correspond the number of elements measured for each parameter.

	Bray Call						Chirps	
	Squeak (SQ)		Grunt (GR)		POP			
	Mean ± SD	n	Mean ± SD	n	Mean ± SD	n	Mean ± SD	n
Duration (s)	0.167 ± 0.093	6	0.248 ± 0.124	7	0.42 ± 0.109	3	0.184 ± 0.111	6
Min. Freq (kHz)	0.629 ± 0.354	6	0.668	1	-	-	3.047 ± 1.31	6
Max Freq (kHz)	1.375 ± 0.566	6	13.131 ± 4.859	7	-	-	8.015 ± 4.801	6
Peak Freq (kHz)	3.891 ± 2.072	6	5.91 ± 0.972	4	14.667 ± 2.309	3	5.695 ± 0.353	4

## Data Availability

The datasets generated and analyzed during the current study are available from the corresponding author upon reasonable request. All fieldwork was conducted under permits issued by the Ministerio de Ambiente de la Provincia de Buenos Aires and in coordination with protected area staff, ensuring compliance with legal and ethical requirements.

## Supplementary Materials

The following supporting information can be downloaded at: https://www.mdpi.com/article/10.3390/ani16050822/s1, Audio S1: Raw audio file (6209.250925112500.wav) of Lahille’s bottlenose dolphins (*Tursiops gephyreus*) recorded in the Bahía Blanca Estuary, Argentina, using a SoundTrap autonomous hydrophone with a sampling rate of 576 kHz. This specific recording was selected for analysis due to its high signal-to-noise ratio (SNR) and the presence of clearly defined echolocation click trains. A total of 1083 clicks were extracted from this file for the parameter analysis presented in this study; Table S1: Results of the generalized additive model (GAM) evaluating diel and tidal patterns in acoustic detections of Lahille’s bottlenose dolphins.
