# Supplementary material for: First Report on the Acoustic Signals of Lahille’s Bottlenose Dolphins in Argentina
Source: Animals (Basel). 2026 Mar 6;16(5):822. doi: 10.3390/ani16050822 (PMC12984323; doi:10.3390/ani16050822)
Supplement: Supplementary file 1 [file animals-16-00822-s001.zip › animals-4104091-supplementary Table S1.pdf]

animals

MDPI

Communication

First Report on the Acoustic Signals of Lahille's Bottlenose Dolphins in Argentina

Gisela Giardino [1,2,*], Agustina Macchi [1], Margherita Silvestri [3], Franck Malige [2,4], Ricardo Bastida [1], Mauricio Soto-Gamboa [3], Iván A. Hinojosa [5,6,7,8], Diego Rodríguez [1], Ignacio Rabinovich [1], Herve Glotin [2,4] and Julie Patris [2,4,9]

Table S1. Results of the generalized additive model (GAM) evaluating diel and tidal patterns in acoustic detections of Lahille's bottlenose dolphins

Table S1. Summary of the generalized additive model (GAM) fitted to evaluate the relationship between dolphin detection-positive minutes per hour (DPM) and hour of day and tidal height. The model was specified as log(E[DPM])=β0+s(Hour of day)+s(Tidal height) and fitted assuming a Poisson error distribution with a log link function. Parameters were estimated using restricted maximum likelihood (REML). Hour of day was treated as a circular variable and modelled using a cyclic cubic regression spline, whereas tidal height was included as a continuous smooth term. The basis dimension (k) was set to 10 for all smooth terms and evaluated using diagnostic checks (gam.check). The model was fitted to 145 hourly observations and explained 72.1% of the deviance in DPM (adjusted R^2 = 0.57). Hour of day and tidal height showed a weak correlation (Pearson's r = 0.20).

Smooth term	edf	Ref.df	Chi-square	p-value
s(Hour of day)	6.58	8.00	14.85	0.024
s(Tidal height)	8.59	8.88	30.11	<0.001

 https://doi.org/10.3390/xxxxx
